# Effects of low-dose organic trace minerals on performance, mineral status, and fecal mineral excretion of sows

**DOI:** 10.5713/ajas.18.0861

**Published:** 2019-04-15

**Authors:** Lianxiang Ma, Junna He, Xintao Lu, Jialing Qiu, Chuanchuan Hou, Bing Liu, Gang Lin, Dongyou Yu

**Affiliations:** 1 College of Animal Science, Zhejiang University, Key Laboratory of Animal Nutrition and Feed in East China of Ministry of Agriculture, Hangzhou, Zhejiang 310058, China; 2 Institute of Quality Standards and Testing Technology for Agricultural Products, Chinese Academy of Agricultural Sciences, Beijing 10081, China

**Keywords:** Organic Trace Mineral, Reproductive Performance, Mineral Status, Fecal Mineral Excretion, Sow

## Abstract

**Objective:**

To investigate the effects of low-dose trace mineral proteinates on reproductive performance, mineral status, milk immunoglobulin contents and fecal mineral excretion of sows.

**Methods:**

Eighty crossbred sows (Landrace×Large White) were randomly allocated to two groups in a 135-day trail, from breeding through 21 d postpartum. The two treatments were inorganic trace minerals (ITM): a basal diet+inorganic iron (Fe), copper (Cu), manganese (Mn), and zinc (Zn) at 90, 15, 25 and 90 mg/kg, respectively and organic trace minerals (OTM): a basal diet+proteinates of Fe, Cu, Mn, and Zn at 72, 12, 20, and 72 mg/kg, respectively.

**Results:**

Compared with ITM, OTM significantly increased (p<0.05) the number of piglets with birthweight >1 kg, the litter weaning weight, and milk Fe, Cu contents. No significant differences (p>0.05) were observed on sow hair mineral contents or immunoglobulin M (IgM), IgG, and IgA contents in colostrum and milk. In comparsion to ITM, OTM decreased fecal Fe, Cu, Mn, and Zn contents of gestating sows (p<0.01) and Fe, Mn, and Zn in lactating sows (p<0.01).

**Conclusion:**

These results indicate that low-dose mineral proteinates can increase the number of piglets with birthweight >1 kg, the litter weaning weight and certain milk mineral concentrations while reducing fecal mineral excretion.

## INTRODUCTION

Fetal growth and development rely entirely on the maternal supply of nutrients, including trace mineral elements [1]. Mineral deficiency in the fetus induced by inadequate transfer of these minerals from the maternal system can result in poor growth and health of the conceptus with negative effects continuing well into the neonatal period [2]. Research on the nutrition of pregnant and lactating sows has been carried out to improve the quality of newborn piglets and maintain good nutritional status of sows throughout their reproductive lifespan [3]. According to Mahan et al [4], a large amount of minerals is transferred from the sow to the fetus during pregnancy, indicating the need to increase supplementation of trace minerals to recover possible depletion. Therefore, it is a common practice, under commercial conditions, to increase the dietary concentrations of supplemental minerals above National Research Council (NRC) [5] recommendations to meet the sows’ needs. When conventional inorganic oxides and sulfates (e.g., ZnO, CuSO_4_) in feed break down in the stomach, the released ions are free to interact with ligands, which will either allow them to remain soluble in the intestine or bind them to insoluble chelates, like phytate, and form low solubility salts which the animal cannot absorb. Under this condition, only a small amount of dietary iron (Fe), zinc (Zn), copper (Cu), and manganese (Mn) will be utilized with 70% to 95% excreted into the environment through manure, which may lead to bioaccumulation in the soil and potentially threaten water sources due to runoff [6,7]. Organic trace minerals (mineral chelate/complex) with stable five- or six-ring structures can be protected from negative interactions with dietary antinutritional factors, remaining electrically neutral at the acidic pH conditions in the digestive tract, thus allowing them to be absorbed and circulated to target positions efficiently [8,9].

Dietary supplementation with organic microminerals has been reported to enhance sow reproductive performance [10], improve micromineral output in mature milk of sows, increase the quantity of mineral transferred across the placenta to the fetus [11,12], and reduce fecal mineral excretion [9]. Hence, low-dose organic trace minerals may replace excess inorganic minerals, satisfying the need of sows while lowering pollution. As most research about organic trace elements in swine has focused on replacing one trace element at NRC [5] recommended levels, data on totally replacing inorganic trace minerals by the organic forms at supplemental levels recommended by commercial industrial production is limited. Therefore, the current experiment was conducted to investigate the effects of totally replacing inorganic Fe, Zn, Cu, and Mn by low-dose organic proteinates on reproductive performance, mineral status, milk immunoglobulin contents and fecal mineral excretion of sows during gestation and lactation.

## MATERIALS AND METHODS

### Animal care

The experimental use of animals and procedures for their management and collection of tissues was performed in accordance with the Chinese Guidelines for Animal Welfare and approved by the Institutional Animal Care and Use Committee of Zhejiang University (Hangzhou, Zhejiang, China).

### Experimental design and treatments

A total of 80 crossbred sows (Landrace×Large White) with similar body weight, parity and breeding date were randomly allocated to 2 treatments with 40 replicates each. Experimental diets were fed for 135 d, from breeding through 21 d postpartum. The two treatments were as follows: inorganic trace minerals (ITM): a basal diet+inorganic Fe, Cu, Mn, and Zn at levels of 90, 15, 25, and 90 mg/kg, respectively in gestation and lactation periods and organic trace minerals (OTM): a basal diet+organic Fe, Cu, Mn, and Zn at levels of 72, 12, 20, and 72 mg/kg (equivalent to 80% of ITM), respectively in gestation and lactation periods. The organic Fe, Cu, Mn, and Zn were metal proteinates, sequestered with enzymatically hydrolyzed soybean protein (Bioplex, Alltech Inc., Nicholasville, KY, USA), the analyzed values of which were 16.7%, 10.2%, 15.8%, and 16.2%, respectively. The inorganic forms of these minerals were provided as FeSO_4_·7H_2_O, Cu_2_(OH)_2_Cl, MnSO_4_·H_2_O, ZnSO_4_·7H_2_O (Weimeng feed Inc., Jiaxing, Zhejiang, China).

Diets of sows were formulated to meet or exceed NRC [5] nutrient requirements. Fe, Cu, and Mn exceeded the NRC [5] requirement, and Zn was on the limit. The ingredients and chemical composition of the basal diets are presented in Table 1. The analyzed values of trace mineral levels in the diet are shown in Table 2.

### Animal housing

During pregnancy sows were housed in individual gestation crates (1.2 m^2^) in an enclosed facility with temperatures averaging 21°C±4°C and fed 3.0 kg feed daily. At approximately 107 d post-coitum sows were placed in individual farrowing crates (1.25 m^2^, hardened steel rod construction) and fed with the lactation treatment diet until weaning at 21 d postpartum. Sows were not fed on the day of farrowing, then the feed amount was raised by 1.0 kg daily until *ad libitum* feeding was reached. Piglets and sows had free access to water via a stainless-steel nipple waterer. Within 3 d postpartum, all piglets were processed (i.e., ear notched and teeth clipped), injected with 200 mg of Fe (iron dextran), and the litter size was equalized between sows within treatments.

### Sampling and processing

Fecal samples from 6 sows per treatment were collected three times daily on d 60 of gestation and day of parturition, then mixed, homogenized and pooled per sow. Each sample was dried in an oven at 65°C for 48 h, then finely ground in a stainless blade grinder to pass a 1 mm screen. Meanwhile, the same sows were selected at the same day to collect approximately 2 to 4 g hair along the topline of the shoulder. The hair was washed with neutral detergent, soaked in acetone for 5 min and rinsed in double distilled water 3 times, then dried for 24 h at 95°C and cut into 0.5 cm lengths to obtain a homogeneous sample [13]. Colostrum and milk were collected (n = 6 per treatment) by hand expression from all functional mammary glands on d 0 and 10 postpartum. The selected sows were each injected with 1 mL oxytocin 10 min prior to milking, and followed by a warm water wash of the udder. Great care was taken to completely milk all the mammary glands to get milk samples of approximately 20 mL. About 10 mL of each sample was centrifuged to collect the whey with an ultracentrifuge at 3,000×g at 4°C for 30 min to remove free fat [14]. All whey, colostrum and milk samples were frozen at −20°C for later analysis.

Piglets were weighed within 2 h of birth, the litter size of total born, born alive, the stillborn and mummies were recorded. On weaning at d 21, the litter size and litter weight were recorded likewise. The weights of dead piglets were documented throughout the entire lactation to calculate average daily gain (ADG) and pre-weaning livability of piglets. On farrowing and weaning day, the backfat thickness of sows at 6 cm perpendicular to the dorsal midline of the last rib was measured using Lean meter (Renco, NY, New York, USA). Feed intake of sows during lactation was recorded daily.

### Analytical methods

Diets, feces and hair were analyzed for Fe, Cu, Mn, and Zn concentrations via an inductively coupled plasma optical emission spectrometer (ICP-OES) (Perkin Elmer Scientific Inc., Billerica, MA, USA) after wet ashing with 3 mL nitric acid and 1 mL hydrogen peroxide in a microwave digester (CEM Corp., Matthews, NC, USA; method 999.10; AOAC [15]). A special alkaline solution was used to process milk samples: 1 g ethylene diamine tetraacetic acid and 10 mL ammonia solution were added to 50 mL double distilled water, agitated, and followed by 10 mL isopropanol and 1 mL 1% Triton X-100 aqueous solution and double distilled water was used to made up to 100 mL. Colostrum and milk samples were thawed at room temperature. Then 0.2 mL of each milk sample was mixed with 0.5 mL of the strong alkaline solution, then vortex mixed for 5 s and made up to a volume of 5 mL with double distilled water [16]. All samples were introduced into the inductively coupled plasma mass spectrometry (ICP-MS) (Perkin Elmer Scientific Inc., USA).

The concentrations of immunoglobulin G (IgG), IgA, and IgM were determined using enzyme-linked immunosorbent assay kits (Jiangsu Mei Biao in Technology Co., Ltd. Yancheng, Jiangsu, China).

### Statistical analysis

Statistics analysis was performed with SPSS 22.0 Software (SPSS, Inc., Chicago, IL, USA). The data were analyzed by T-test and the results are presented as mean and standard error of mean. Significance was declared at p<0.05, and p<0.10 was considered to represent tendencies between treatments.

## RESULTS

### Reproductive performance

Table 3 shows the reproductive performance of sows fed different sources of trace minerals. Compared with ITM, OTM increased (p<0.05) the number of piglets with birth weight >1 kg by 6.5%. Litter weaning weight was increased 15.8% (p<0.05) with OTM. However, there were no significant differences for litter size of total born, born alive, litter birth weight, weaning survival rate or ADG of piglets between the two treatments.

### Milk trace minerals contents

OTM increaed milk Fe and Cu contents (p<0.01) and tended to increase colostrum Zn content (p = 0.09) compared with ITM (Table 4).

### Immunoglobulin contents

Table 5 shows that there were no significant differences in colostrum and milk IgA, IgG, and IgM contents from sows fed diets supplemented with ITM or OTM.

### Hair and fecal trace minerals concentrations

As shown in Table 6, compared with ITM, OTM significantly decreased (p<0.01) Fe, Mn, Cu, and Zn contents in the manure of gestating sows by 33.5%, 15.6%, 77.3%, and 37.8%, respectively. Fecal Fe, Mn, and Zn contents were reduced during lactation by 47.7%, 28.8%, and 56.1%, respectively (p<0.01). However, the contents of Fe, Cu, Mn, and Zn in sows’ hair throughout gestation and lactation had no distinct differences between the treatments.

## DISCUSSION

During the gestation and lactation periods, sows have an increased demand for trace elements such as Fe, Cu, Mn, and Zn to support their growth and production needs. Fetal growth and development rely entirely on the maternal nutrient supply, including trace minerals, deficiency of which will result in poor growth and health of the conceptus [1,2]. It has been reported that iron amino acid chelates could increase the proportion of iron transferred to fetus through the placenta and obtain higher piglet growth rate [12,17], and that Cu proteinate could reduce the weight loss of lactating sows while improving the litter weaning weight [18]. In addition, organic trace minerals complexes (Cu, Fe, Mn, Zn, and Se) increased the number of pigs born (total and live), litter birth weight [10] and individual piglet weight at birth and at weaning [3]. In contrast, Acda and Chae [19] demonstrated that organic trace elements produced no effect on the number of piglets total born, born alive and weaned, which was in accordance with the results of our study, this could result from the complexity of the female reproductive process. However, it was observed in the present study that compared with ITM, sows fed the OTM diet had more piglets with birthweight >1 kg, which is a crucial indicator in commercial pig production.

Serum and tissues are commonly used to reflect body mineral status [11,20]. However, in the current study, hair of sows was collected to determine Fe, Cu, Mn, and Zn concentrations instead of serum in consideration of the negative effect on the conceptus induced by blood sampling stress. Moreover, hair had been reported as an appropriate alternative for analysis of minerals in humans [21] and swine [22], as it could be readily obtained, stored and reflect the average load of minerals in the body at a particular time. In the present study, no differences were observed in hair mineral contents of sows during gestation and lactation when fed ITM or OTM, indicating that organic trace minerals at 80% of ITM levels may be sufficient for internal absorption and utilization by commercial sows. Colostrum and milk are known to provide nutritional support to neonates to aid their development, and the trace minerals in them may be influenced by diet composition and bioavailability due to transfer from plasma to the mammary gland [8]. Some studies have shown that Fe and Cu contents in milk are generally resistant to dietary levels, whereas Zn and Mn concentrations in milk increased with increasing dietary levels in the dam [23]. Interestingly, Peters and Mahan [11] observed a linear relationship between milk Zn and Cu contents and dietary supplementation levels. In the current study, milk Fe and Cu increased and colostrum Zn content tended to increase with dietary supplementation of organic trace minerals complexes, which was consistent with the results of Acda and Chae [19] and Bertechini et al [3]. This implies that organic Fe and Cu can be more efficiently utilized and transferred to mammary glands, possibly resulting in heavier litter weight. One possible interpretation is that organic Fe can be transferred more effectively from placenta to the fetus [12]. The above results indicate that organic trace minerals have higher bioavailability and can exert the same or better growth-promoting effect than higher levels of inorganic forms.

Newborn piglets with immature immune systems rely entirely on colostrum immunoglobulins, mainly IgG to provide passive immunity. As reported by Kielland et al [24], increasing IgG level in colostrum would improve the level in piglets and potentially enhance preweaning survival. Furthermore, the sensitivity of neonatal piglets to pathogen infection can be reduced by immunoglobulins in colostrum and milk [14, 25]. Ig A is a major immunoglobulin in milk that helps protect piglets against pathogenic bacteria, commensal bacteria and food antigens in the local digestive tract [14]. Trace minerals (Fe, Zn, and Mn) are vital for the formation and stabilization of immune system as enzyme co-factors and are involved in the regulation of immune cell repair, proliferation, and gene transcription, deficiency of which may affect normal immune function and reduce serum immunoglobulin contents [26]. Wu et al [27] reported that zinc amino acid chelates increased the contents of IgG, IgA, and IgM in serum of weaned pigs and lymphocyte transformation rate. Iron amino acid chelate was also observed to elevate IgG level in suckling pigs’ serum [28]. Moreover, pregnant sows fed with organic trace minerals complexes (Fe, Zn, Mn, Se, and Cr) considerably improved serum IgG levels [26]. In this study, no differences were observed on the contents of IgG, IgM, or IgA in the colostrum and milk of sows, implying that OTM at 80% of ITM levels was sufficient, having the same effect as 100% ITM in allowing lactating sows to secrete immunoglobulins.

Minerals in excrement are mainly derived from unabsorbed trace minerals in the diet and those excessively absorbed and excreted by the bile under the action of homeostatic balance regulation [20]. Thus, the supplemental levels and sources of trace minerals in the diet are the main factors affecting micromineral emissions in excrement [29,30]. Dietary supplementation with low levels of trace minerals has been reported to reduce micromineral concentrations in the manure of nursery pigs [30] and grower-finisher pigs [20]. Moreover, using organic trace elements totally or individually to replace the inorganic trace minerals also decreased fecal trace elements excretion [30,31]. However, there are few reports of the fecal micromineral excretion of gestating and lactating sows when inorganic Fe, Cu, Mn, and Zn was totally replaced by organic trace elements at low levels. Results from the present study showed that reducing organic trace minerals (Fe, Cu, Mn, and Zn) supplementation to 80% of the commercially recommended levels reduced fecal Fe, Cu, Mn, and Zn excretion by 25.1%, 13.5%, 43.7%, and 27.4%, respectively in gestating sows. While fecal Fe, Mn, and Zn concentrations were reduced by 32.3%, 22.3%, and 35.9%, respectively in lactating sows. These reductions occurred without adverse effects on performance. Similarly, our team also found, in previous work, that fecal contents of Fe, Cn, Zn, and Se were significantly decreased when growing-fattening pigs consumed organic trace mineral compounds (Fe, Cn, Mn, Zn, and Se) or were fed diets without trace elements inclusion in comparison to those receiving equivalent inorganic trace minerals at commercially recommended levels without compromising growth performance [20]. The possible interpretation could be that organic trace minerals with stable five- or six-membered ring structures can be more completely absorbed by the intestinal brush border through amino acid or peptide absorption routes and protected from interference in digestive tract from compounds such as phytate and calcium, resulting in higher bioavailability and lower fecal mineral excretion [18]. These routes of absorption need further exploration to fully explain the ability to feed organic trace minerals at low levels without compromising performance.

In summary, total replacement of inorganic trace element compounds (Fe, Cu, Mn, and Zn) by mineral proteinates at approximately 80% increased the number of piglets with birthweight >1 kg, litter weaning weight, and milk mineral levels and reduced trace mineral excretion of commercial gestating and lactating sows, while having no significant influences on immunoglobulins IgG, IgA, and IgM levels in colostrum and milk of the sows.

## Figures and Tables

**Table 1:** Composition and nutrient levels of basal diets (as-fed basis, %)

Items	Pregnancy	Lactation
Ingredients		
Corn	59.30	63.00
Wheat	6.00	-
Soybean meal	19.50	23.50
Extruded soybean	-	3.00
Fish meal	1.00	2.00
Wheat middling	4.00	4.00
Rice bran	6.70	-
soybean oil	-	0.75
Limestone	1.35	1.20
Dicalcium phosphate	0.80	1.20
Salt	0.35	0.35
Premix1)	1.00	1.00
Total	100.00	100.00
Calculated composition		
Digestible energy (MJ/kg)	13.61	13.94
Crude protein (%)	16.55	18.55
Lysine (%)	0.79	0.95
Methionine (%)	0.27	0.30
Threonine (%)	0.61	0.70
Calcium (%)	0.87	1.01
Total phosphorus (%)	0.63	0.68
Available phosphorus (%)	0.34	0.47

1) Supplemented per kg of diet: vitamin A, 10,000 IU; vitamin D_3_, 3,000 IU; vitamin E, 50 IU; vitamin K_3_, 2 mg; vitamin B_1_, 2 mg; riboflavin, 4.8 mg; vitamin B_6_, 3 mg; vitamin B_12_, 20 μg; niacin, 20 mg; folic acid, 3 mg; pantothenic acid, 12 mg; choline hydrochloride, 300 mg; Se, 0.3 mg; I, 1.5 mg.

**Table 2:** Analyzed value of trace mineral levels in the diets (as-fed basis, mg/kg)

Trace minerals	Treatments1)	

	ITM	OTM
Gestating feed		
Fe	304.2 (90)	283.1 (72)
Cu	29.5 (15)	25.6 (12)
Mn	67.4 (25)	61.7 (20)
Zn	129.4 (90)	116.5 (72)
Lactating feed		
Fe	321.5 (90)	302.1 (72)
Cu	34.7 (15)	29.9 (12)
Mn	64.4 (25)	60.9 (20)
Zn	118.5 (90)	103.8 (72)

Values in parenthesis are the amounts of added minerals.

1) ITM, inorganic trace minerals; OTM, organic trace minerals.

**Table 3:** Effect of organic trace minerals on reproductive performance of sows

Parameters	ITM1)	OTM1)	SEM	p-value
Parity (n)	2.58	2.65	0.18	0.77
Back fat thickness at farrowing (mm)	16.39	16.56	0.60	0.85
Back fat thickness at weaning (mm)	14.97	15.19	0.48	0.76
Lactation feed intake (kg/d)	5.63	5.65	0.04	0.73
Total born (n)	11.75	11.81	0.22	0.90
Born alive (n)	11.05	11.28	0.19	0.55
Piglets with birth weight >1 kg (n)	9.83	10.47	0.15	0.03
Stillborn and mummies (n)	0.75	0.56	0.11	0.38
Litter birth weight (kg)	15.72	16.69	0.31	0.12
Piglet birth weight (kg)	1.43	1.49	0.02	0.18
Pigs at weaning (n)	9.52	10.45	0.47	0.17
Litter weaning weight (kg)	59.41	68.77	2.82	0.02
Piglet weaning weight (kg)	6.48	6.72	0.23	0.48
Weaning survival rate (%)	86.34	91.33	2.46	0.15
1 to 21 d ADG (g/d)	214.57	229.23	9.92	0.25

Values are presented as mean and SEM; n = 40.

SEM, standard error of the mean; ADG, average daily gain.

1) ITM, inorganic trace minerals; OTM, organic trace minerals at 80% of ITM.

**Table 4:** Effect of organic trace minerals trace minerals contents in sows’ milk (μg/mL)

Items	ITM1)	OTM1)	SEM	p-value
Parity (n)	2.67	2.50	0.21	0.60
Backfat thickness (mm)	16.23	16.33	0.50	0.90
Lactation feed intake (kg/d)	5.64	5.65	0.03	0.75
Colostrum				
Fe	13.69	13.57	1.37	0.96
Mn	0.06	0.05	0.01	0.29
Cu	4.32	3.44	0.68	0.42
Zn	15.95	23.41	3.16	0.09
Milk				
Fe	12.39	18.06	2.11	0.03
Mn	0.09	0.14	0.03	0.22
Cu	0.86	1.53	0.23	0.01
Zn	8.79	8.20	0.76	0.64

Values are presented as mean and SEM; n = 6.

SEM, standard error of the mean.

1) ITM, inorganic trace minerals; OTM, organic trace minerals at 80% of ITM.

**Table 5:** Effect of organic trace minerals on immunoglobulin contents in sows’ milk (μg/mL)

Items	ITM1)	OTM1)	SEM	p-value
Colostrum				
IgM	113.26	123.13	5.48	0.46
IgG	426.15	447.87	17.62	0.58
IgA	118.51	121.41	6.24	0.74
Milk				
IgM	101.67	107.33	4.88	0.58
IgG	366.12	321.29	11.76	0.10
IgA	134.40	129.08	5.20	0.70

Values are presented as mean and SEM; n = 6.

SEM, standard error of the mean; Ig, immunoglobulin.

1) ITM, inorganic trace minerals; OTM, organic trace minerals at 80% of ITM.

**Table 6:** Effect of organic trace minerals on trace minerals contents in hair and feces of sows (mg/kg, as air-dry basis)

Items		ITM1)	OTM1)	SEM	p-value
Hair	Gestation				
	Fe	19.00	20.64	2.96	0.74
	Mn	3.84	3.21	0.69	0.56
	Cu	15.26	14.03	0.55	0.12
	Zn	358.98	342.30	29.92	0.73
	Lactation				
	Fe	28.47	31.06	1.76	0.35
	Mn	5.99	4.88	0.85	0.39
	Cu	15.68	15.08	0.69	0.57
	Zn	433.52	468.47	30.63	0.45
Feces	Gestation				
	Fe	2,350.04	1,760.90	152.81	<0.01
	Mn	1,051.57	910.06	39.55	<0.01
	Cu	404.64	228.24	42.44	<0.01
	Zn	1,783.92	1,294.82	119.90	<0.01
	Lactation				
	Fe	3,411.54	2,310.22	303.59	<0.01
	Mn	1,199.74	931.76	69.19	<0.01
	Cu	319.78	341.45	11.14	0.19
	Zn	2,330.00	1,492.99	221.31	<0.01

Values are presented as mean and SEM; n = 6.

SEM, standard error of the mean.

1) ITM, inorganic trace minerals; OTM, organic trace minerals at 80% of ITM.
